# Nickel-Catalyzed Cyanation of Aryl Halides

**DOI:** 10.3390/molecules30163440

**Published:** 2025-08-20

**Authors:** Zhenqiang Ma, Cuimeng Huo, Duo Zhou, Jingyi Zhang, Hongjun Kong, Wenke Ren, Fengbo Qu, Tingting Liu, Hui Chen, Xilong Wang

**Affiliations:** 1 Henan Province Key Laboratory of Environmentally Friendly Functional Materials, Institute of Chemistry, Henan Academy of Sciences, Zhengzhou 450002, China; 2 College of Chemistry, Zhengzhou University, Zhengzhou 450001, China

**Keywords:** nickel catalysis, cyanation, aryl halides

## Abstract

Nickel-catalyzed cyanation of aryl halides has emerged as a powerful and sustainable method for the synthesis of aryl nitriles—valuable motifs widely found in pharmaceuticals, agrochemicals, and functional materials. Compared to traditional cyanation methods that involve harsh conditions and toxic reagents, nickel catalysis enables mild, efficient, and versatile transformations. This review systematically summarizes recent advances in this field, categorized by different cyanide sources, including metal cyanides (NaCN, KCN, Zn(CN)_2_, K_4_[Fe(CN)_6_]) and non-metal or organic cyanide sources (e.g., MeCN, nitriles, BrCN, CO_2_/NH_3_). Key developments in catalyst systems, ligand design, mechanistic insights, and green chemistry aspects are highlighted. Remaining challenges and future directions are also discussed.

## 1. Introduction

**Scheme 1:**
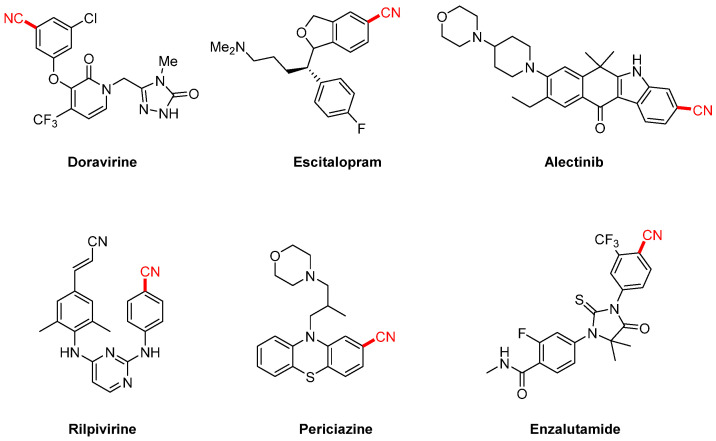
Aryl nitrile-containing pharmaceutical molecules.

Aryl nitriles, as essential structural motifs, are widely found in natural products, pharmaceuticals, and functional materials due to their unique chemical reactivity and excellent synthetic versatility [1,2]. In medicinal chemistry, the aryl nitrile group is frequently associated with significant biological activity [3,4,5]. For instance, several therapeutically important drugs, such as the non-nucleoside reverse transcriptase inhibitor doravirine (used in HIV treatment), the antidepressant escitalopram, and the antipsychotic pipamperone, all feature nitrile functionalities. Moreover, the introduction of a cyano group can significantly enhance the physicochemical and pharmacological properties of drug molecules [6,7]. For example, incorporating a nitrile group into farnesyltransferase inhibitors markedly improves their pharmacokinetic profile, increases water solubility by nearly 10-fold compared to brominated analogs, and boosts biological activity by up to 44-fold. In sulfonamide derivatives, the cyano group effectively reduces molecular basicity and significantly enhances antibacterial activity (Scheme 1). Beyond pharmaceutical applications, aryl nitriles also serve as versatile intermediates for the synthesis of high-value compounds such as aldehydes, ketones, amides, amines, and carboxylic acids through transformations including hydrolysis, reduction, and nucleophilic addition, thereby demonstrating their broad synthetic utility [8,9,10,11,12].

The synthesis of aryl nitriles dates back to the 19th century, with classical approaches including the Sandmeyer reaction [13,14] and the Rosenmund–von Braun reaction [15,16,17]. The former involves the diazotization of aryl amines followed by treatment with CuCN to generate aryl nitriles, whereas the latter proceeds via direct reaction of aryl iodides with excess CuCN at elevated temperatures (≥150 °C). In industrial settings, aryl nitriles are typically synthesized by reacting toluene derivatives with ammonia and oxygen at 300–550 °C [18]. Despite their historical significance, these traditional methods often suffer from harsh reaction conditions, large amounts of heavy metal waste, and serious environmental concerns. With the rapid advancement of transition metal catalysis, particularly involving palladium, copper, and nickel, the synthesis of aryl nitriles has evolved toward more efficient and environmentally benign methodologies. Recent developments have introduced diverse catalytic strategies, including cyanation of aryl halides, deoxycyanation of phenol derivatives [19,20,21,22,23,24,25], direct C–H cyanation of arenes [26,27,28,29,30,31,32], and other innovative nitrile-forming transformations [33,34]. Among these, cyanation of aryl halides has gained significant attention due to its operational simplicity, mild reaction conditions, and broad substrate scope.

**Scheme 2:**
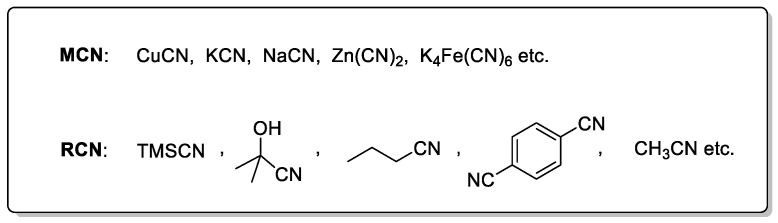
Common cyanating reagents.

The successful incorporation of a cyano group heavily relies on the selection of an appropriate cyanide source, which is generally classified into two major categories: metal cyanides and organic cyanating reagents. Common metal-based sources include NaCN [35,36,37,38,39,40], KCN [41,42,43,44,45,46,47,48], Zn(CN)_2_ [49,50,51,52,53,54,55], and K_4_[Fe(CN)_6_] [56,57]. Among them, NaCN and KCN are highly toxic and pose serious safety concerns, while Zn(CN)_2_ is less toxic and poorly soluble, which helps reduce the risk of catalyst poisoning. Notably, K_4_[Fe(CN)_6_] is virtually non-toxic, thermally stable, and operationally safe, making it widely applicable in both laboratory and industrial settings (Scheme 2). On the other hand, organic cyanide sources include trimethylsilyl cyanide (TMSCN) [58,59], acetone cyanohydrin [60], aliphatic and aromatic nitriles, and benzyl cyanide [61]. Certain solvents such as acetonitrile [62,63,64] can also serve as cyanide sources under appropriate conditions. In recent years, cyanide-free systems such as those based on DMF and ammonium salts have been developed as greener alternatives [31,65], further broadening the applicability of cyanation reactions. Over the past two decades, palladium-catalyzed cyanation of aryl halides has been extensively investigated and summarized in several comprehensive reviews [66,67]. Compared to palladium, nickel is more cost-effective, more abundant in the Earth’s crust, and possesses unique electronic properties, making the study of nickel-catalyzed cyanation reactions both valuable and necessary. While some progress in nickel-catalyzed cyanation had already been made prior to 2020 [68,69], this review aims to provide a more detailed and up-to-date overview of recent advances in the nickel-catalyzed cyanation of aryl halides. Given the distinct differences in toxicity, reactivity, and substrate compatibility among various cyanide sources, this review focuses on nickel-catalyzed cyanation of aryl halides, categorized and analyzed by the type of cyanide source employed.

## 2. Metal Cyanide Sources

### 2.1. NaCN and KCN

**Scheme 3:**
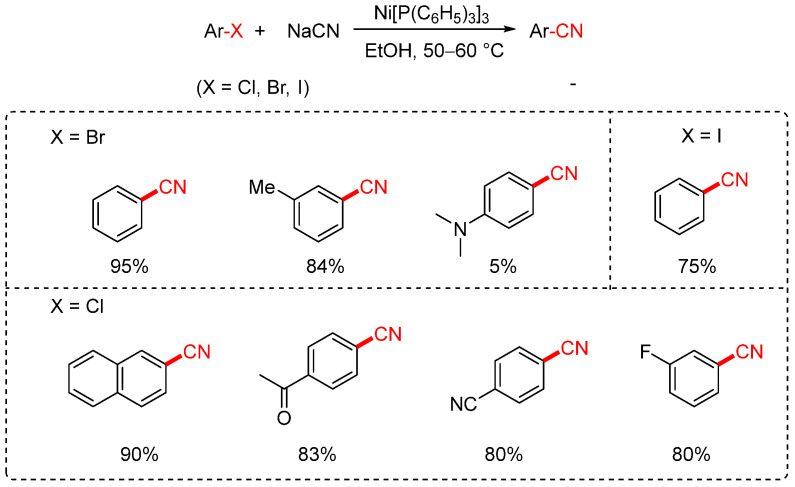
First Ni-catalyzed cyanation of aryl halides (NaCN).

In 1973, the Cassar group reported the first nickel-catalyzed cyanation of aryl halides using sodium cyanide (NaCN) as the cyanating reagent (Scheme 3) [35,70]. The reaction employed Ni(PPh_3_)_3_ as the catalyst and ethanol as the solvent, operating at temperatures between 50 and 60 °C. Although the method enabled the conversion of aryl bromides and iodides to the corresponding aryl nitriles, its substrate scope was rather limited. High yields were achieved only with substrates such as iodobenzene, bromobenzene, and m-bromotoluene, whereas less reactive compounds, such as aniline derivatives, afforded only trace amounts of the desired products. In the case of aryl chlorides, only a few highly activated substrates underwent successful transformation with satisfactory yields.

**Scheme 4:**
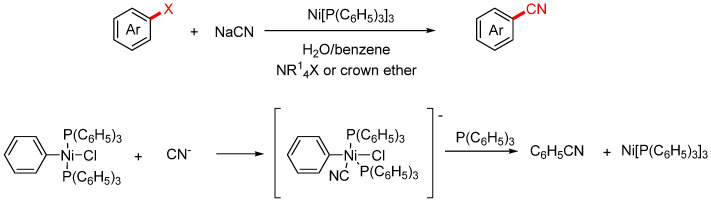
Ni-catalyzed cyanation of aryl halides and the proposed mechanism.

In 1979, the Cassar group developed an improved method for the nickel-catalyzed cyanation of aryl halides [36]. This approach offered two key advantages: high turnover frequency of the catalyst and operational simplicity, making it well-suited for both laboratory-scale synthesis and potential industrial application (Scheme 4). The reaction was carried out in a biphasic water–benzene system, where the aryl halide, ammonium salt or crown ether, and nickel complex were dissolved. An aqueous solution of NaCN was then added dropwise into the system, with the addition rate carefully controlled to avoid catalyst deactivation caused by excess cyanide. Based on systematic mechanistic investigations, the authors proposed the following catalytic cycle: Under Ni(PPh_3_)_3_ catalysis, the aryl halide (Ar–X) undergoes oxidative addition to generate an aryl–Ni(II) intermediate. The cyanide anion (CN^−^), present in the aqueous phase, is transferred into the organic phase with the assistance of a phase-transfer catalyst such as a quaternary ammonium salt or crown ether. It then undergoes ligand exchange with the Ni(II) intermediate, releasing the aryl nitrile product and regenerating the Ni(0) catalyst to complete the catalytic cycle.

**Scheme 5:**
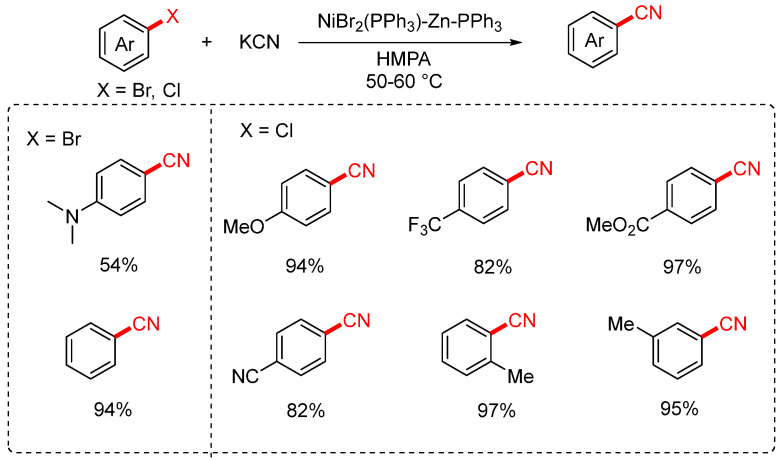
Ni-catalyzed cyanation of aryl halides (KCN/HMPA system).

In 1988, the Takagi group developed a nickel-catalyzed cyanation of aryl halides using potassium cyanide (KCN) as the cyanating agent (Scheme 5) [43,71]. The reaction employed a catalytic system consisting of NiBr_2_(PPh_3_)_2_, zinc, and triphenylphosphine, with HMPA or acetonitrile as the solvent, and was conducted at 40–80 °C. The protocol enabled the transformation of both aryl chlorides and bromides, exhibiting particularly high efficiency for electron-deficient aryl chlorides bearing substituents such as CF_3_, COOMe, and COMe. In contrast, aryl bromides containing electron-donating groups like NMe_2_ gave only moderate yields. Notably, when o-dichlorobenzene was used as the substrate, the reaction afforded the mono-cyanated product in 28% yield and the dicyanated product in 67% yield.

**Scheme 6:**
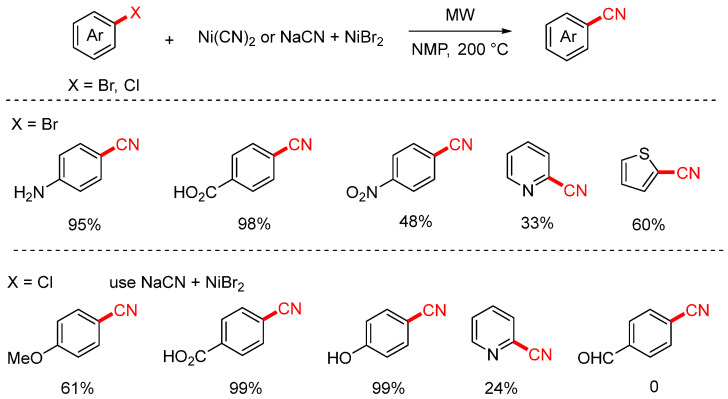
Ni-catalyzed microwave-assisted cyanation of aryl halides (Ni(CN)_2_/NaCN).

In 2003, the Leadbeater group successfully achieved the cyanation of aryl bromides and some aryl chlorides using Ni(CN)_2_ under microwave irradiation conditions (Scheme 6) [72]. This method enabled the transformation of challenging aryl bromides bearing functional groups such as carboxyl, amino, and nitro groups. However, under these conditions, cyanation of aryl chlorides occurs only with very low yields. To improve this, the authors subsequently employed a NaCN/NiBr_2_ system, which allowed for the cyanation of aryl chlorides. Nevertheless, both approaches showed limited efficiency and substrate scope when applied to heteroaryl halides, resulting in generally low yields.

**Scheme 7:**
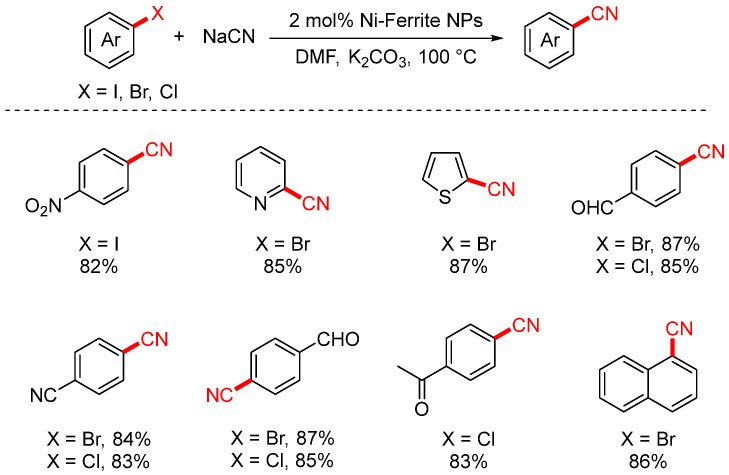
NiFe_2_O_4_-catalyzed cyanation of aryl halides.

In 2014, Rezvani achieved cyanation of aryl halides using bimetallic NiFe_2_O_4_ nanoparticles as the catalyst (Scheme 7) [73]. The use of bimetallic nanoparticles significantly enhanced both selectivity and catalytic activity, and they exhibited stronger resistance to deactivation compared to monometallic nanoparticles. This catalytic system greatly shortened the reaction time (to 17–65 min), and the catalyst could be easily separated and reused after the reaction. Moreover, it substantially improved the yields of 4-nitrobenzonitrile and various heteroaryl nitriles.

### 2.2. Zn(CN)_2_

**Scheme 8:**
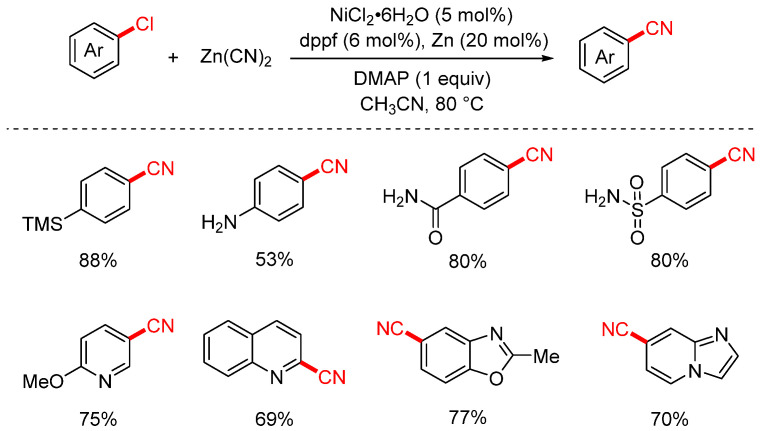
Ni-catalyzed cyanation of (hetero)aryl chlorides (Zn(CN)_2_).

In 2017, Liu Yuanhong’s group first utilized Zn(CN)_2_ as the cyanation reagent to achieve the cyanation of aryl and heteroaryl chlorides (Scheme 8) [74]. The reaction employed Zn as the reductant, NiCl_2_·6H_2_O as the catalyst, dppf as the ligand, acetonitrile as the solvent, and DMAP as an additive. Under mild conditions (80 °C), this system efficiently facilitated the cyanation of a broad range of aryl chlorides. Aryl chlorides bearing functional groups such as methoxy, ester, and acetyl were smoothly converted into the corresponding nitriles in excellent yields. In addition, various heteroaryl chlorides, including pyrazoles, thiophenes, and indoles, were also successfully transformed with good to high efficiency.

**Scheme 9:**
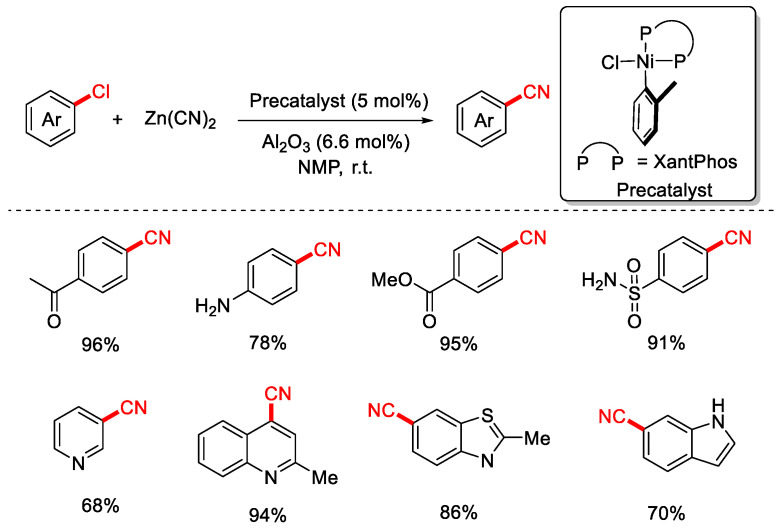
Ni-catalyzed room-temperature cyanation of aryl chlorides (Zn(CN)_2_/Al_2_O_3_).

In 2017, the Beller group employed Zn(CN)_2_ as the cyanating reagent and an air-stable Ni(II)-XantPhos precatalyst to achieve the cyanation of aryl and heteroaryl chlorides under ambient temperature conditions (Scheme 9) [75]. In this system, solid Al_2_O_3_ (derived from AlCl_3_·6H_2_O) was added to promote the reaction, as Lewis acids are known to facilitate the transfer of cyanide from the solid phase to the solution. However, the exact role of Al_2_O_3_ in this transformation remains unclear.

**Scheme 10:**
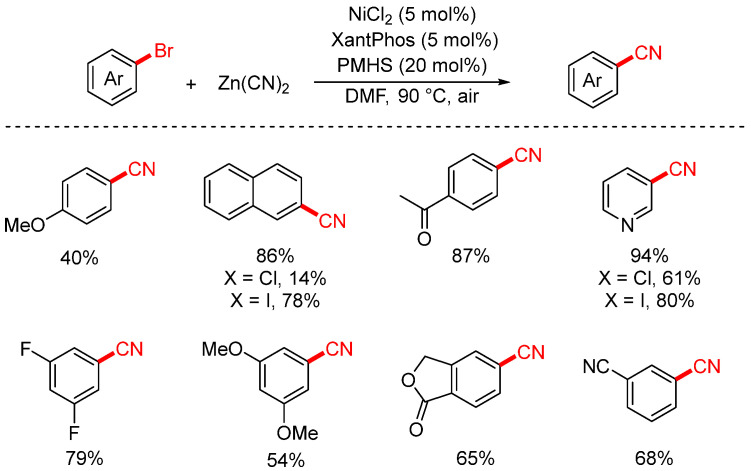
Ni-catalyzed air-tolerant cyanation of aryl chlorides (Zn(CN)_2_/PMHS).

In 2022, the Sifri group achieved the cyanation of aryl halides under air using Zn(CN)_2_ as the cyanating reagent (Scheme 10) [76]. The reaction employed NiCl_2_ as the catalyst, XantPhos as the ligand, and polymethylhydrosiloxane (PMHS) as the reductant, operating efficiently under open-air conditions. By replacing the traditional reductant Zn with the more environmentally friendly PMHS, the overall reaction conditions became greener and more sustainable.

**Scheme 11:**
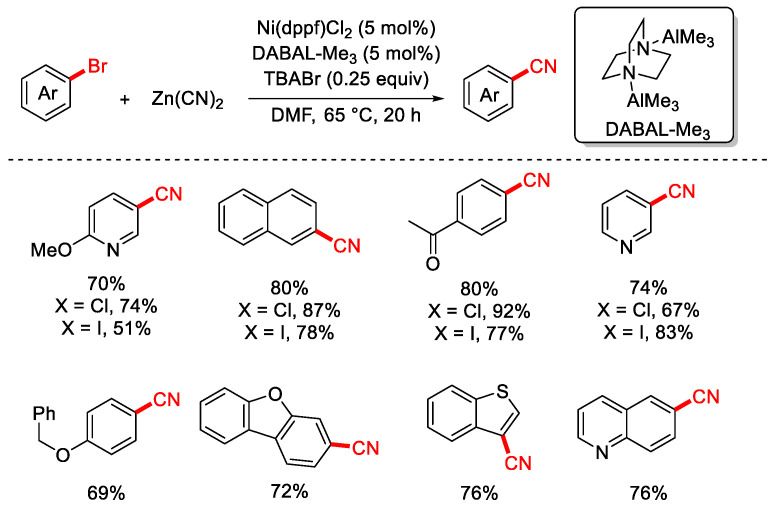
Ni-catalyzed cyanation of aryl chlorides (Zn(CN)_2_/DABAL-Me_3_ reductant).

In 2022, the Hethcox group reported a cyanation of aryl halides using Zn(CN)_2_ as the cyanating reagent (Scheme 11) [77]. The reaction employed air-stable and preactivation-free DABAL-Me_3_ as the reductant, eliminating the need for the preactivation steps typically required when using traditional reductants such as Zn or Mn. Additionally, tetrabutylammonium bromide (TBABr) was added to the system, and control experiments confirmed that it facilitated the dissolution of low-solubility cyanide salts, thereby enhancing the efficiency of the reaction.

**Scheme 12:**
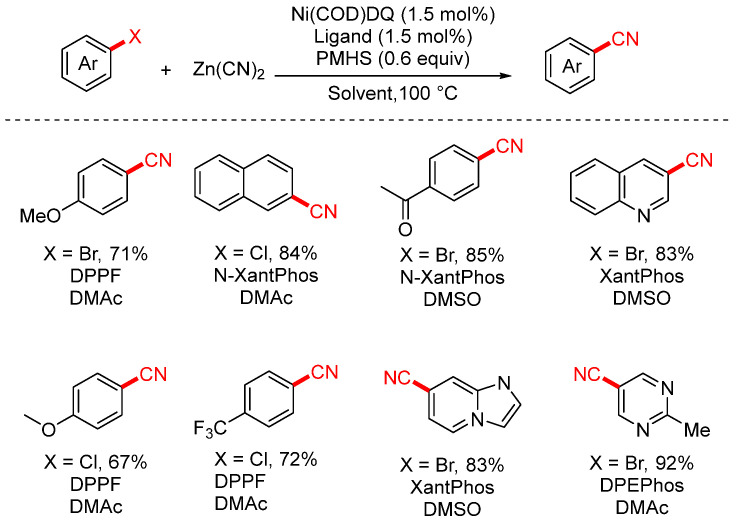
High-throughput screening of Ni-catalyzed cyanation of aryl chloride systems.

In 2025, the Santagostino group reported a novel screening platform for the nickel-catalyzed cyanation of (hetero)aryl halides (Scheme 12) [78]. This method utilized the air-stable Ni(COD)DQ complex as the catalyst and employed high-throughput experimentation (HTE) to systematically explore various ligand and solvent combinations, enabling the rapid identification of optimal reaction conditions.

## 3. Non-Metal or Organic Cyanide Sources

### 3.1. Cyanating Reagents

**Scheme 13:**
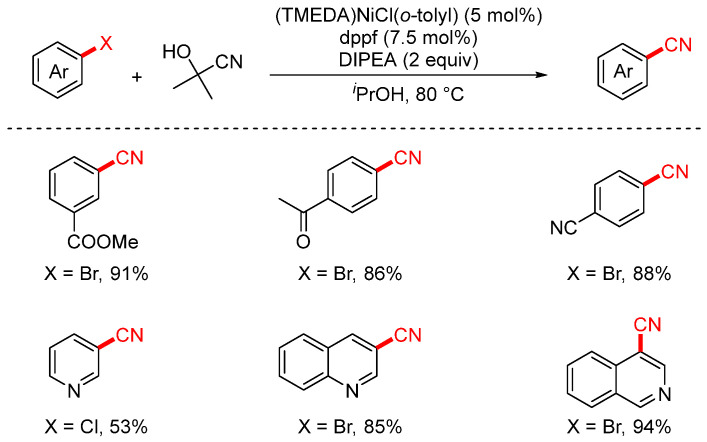
Ni-catalyzed cyanation of aryl chlorides using acetone cyanohydrin.

In 2016, the Guimond group employed acetone cyanohydrin as a cyanating reagent to achieve the nickel-catalyzed cyanation of aryl halides (Scheme 13) [79]. Compared to traditional metal-based cyanide sources, acetone cyanohydrin is less toxic, more cost-effective, and offers higher atom economy. The reaction proceeded efficiently using either [Pd(cinnamyl)Cl]_2_/XPhos or the newly developed (TMEDA)NiCl(o-tolyl) precatalyst, affording the desired products in excellent yields.

**Scheme 14:**
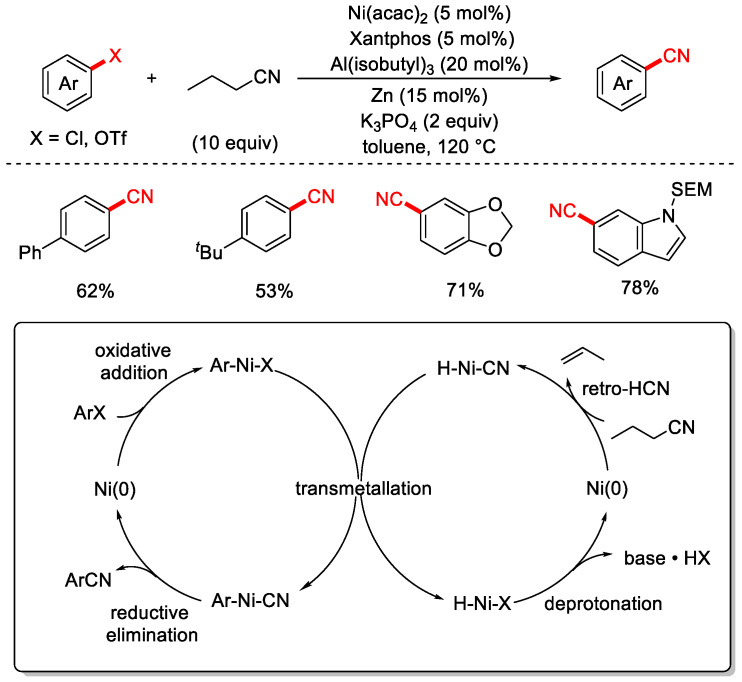
Ni-catalyzed cyanation of aryl chlorides using butyronitrile.

In 2017, the Morandi group was the first to employ a low-toxicity organic cyanide source—nitrile (butyronitrile)—as the cyanating reagent to achieve the cyanation of aryl chlorides (Scheme 14) [80]. The reaction utilized Ni(acac)_2_ as the catalyst, XantPhos as the ligand, Zn as the reductant, and Al(isobutyl)_3_ as an additive. This method enabled both the cyanation of aryl chlorides and the cyanation of aryl triflates (Ar–OTf). For aryl chlorides, the reaction tolerated a broad substrate scope with moderate to good yields, while for aryl triflates, both the substrate range and yields were excellent. The proposed reaction mechanism involves the oxidative addition of Ni(0) to the C–CN bond of butyronitrile, assisted by a Lewis acid, forming a Ni(II)–alkyl–cyano intermediate. A subsequent β-hydride elimination releases an olefin (e.g., propene) and generates a Ni(II)–H–CN species. Meanwhile, oxidative addition of the aryl halide to Ni(0) yields an Ar–Ni(II)–X intermediate. Transmetalation between the two Ni(II) species forms the key Ar–Ni(II)–CN intermediate, which undergoes reductive elimination to furnish the desired aryl nitrile and regenerate Ni(0), completing the catalytic cycle. Additionally, the H–Ni–X species formed via β-hydride elimination is converted back to Ni(0) in the presence of a base.

**Scheme 15:**
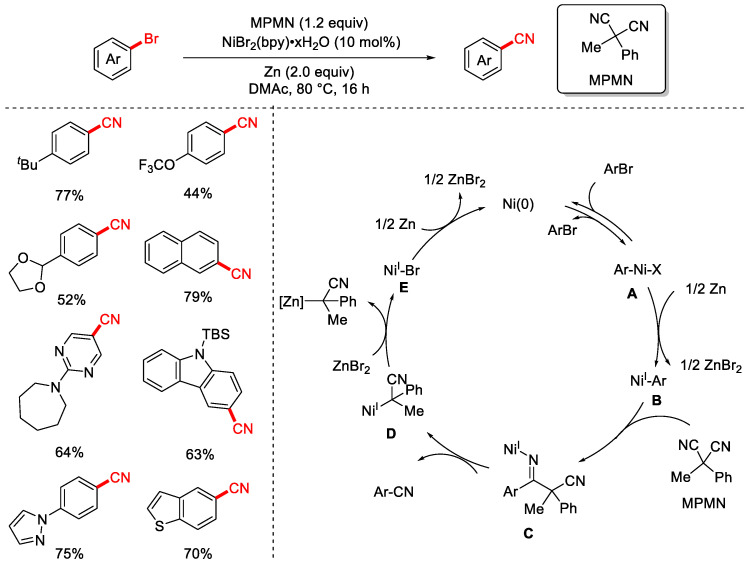
Ni-catalyzed cyanation of aryl bromides using MPMN as a cyano source.

In 2019, the Rousseaux group successfully achieved the cyanation of aryl halides using a stable and low-toxicity organic cyanide reagent, MPMN (Scheme 15) [81]. The reaction employed NiBr_2_(bpy)·xH_2_O as the catalyst and DMAc as the solvent, proceeding at 80 °C for 16 h for efficient cyanation of aryl bromides. The substrate scope was broad, tolerating various substituents and heteroaryl bromides, and the method was also applied to the derivatization of bioactive molecules. When NaBr was added as an additive, the reaction could also achieve dechlorocyanation of aryl chlorides, albeit with a narrower substrate scope and lower yields. The proposed mechanism begins with a Ni(0) intermediate undergoing reversible oxidative addition to the aryl bromide, forming a Ni(II) intermediate A. Subsequent reduction by zinc produces a Ni(I)–aryl intermediate B. Migratory insertion of MPMN via a 1,2-insertion generates a Ni(I)-bound imine intermediate C, which undergoes β-carbon elimination to release the benzonitrile product and an α-nickelated intermediate D. Ligand exchange with ZnBr_2_ then affords the Ni(I)–Br intermediate E, which is reduced by zinc to regenerate the active Ni(0) catalyst, thus completing the catalytic cycle.

**Scheme 16:**
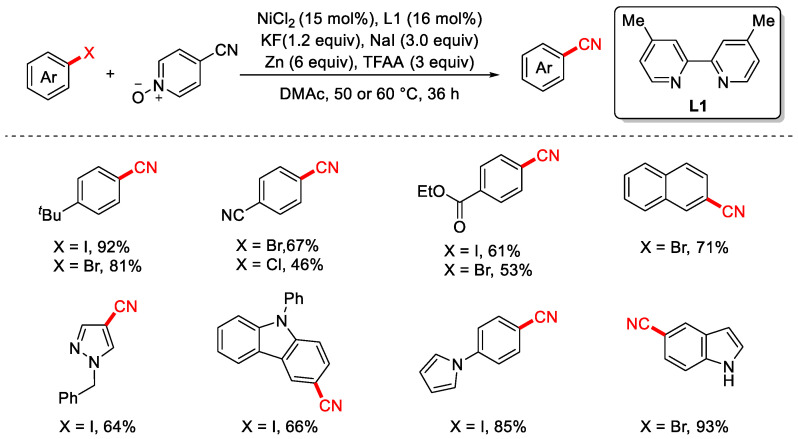
Ni-catalyzed cyanation of aryl halides using 4-cyanopyridine N-oxide.

In 2020, the Liao group reported the cyanation of aryl halides using an inexpensive and non-toxic 4-cyanopyridine N-oxide as the cyanation reagent (Scheme 16) [82]. The reaction proceeds under mild conditions with good functional group tolerance and a broad substrate scope. Notably, the presence of NaI plays a crucial role in suppressing the hydrogenation side reaction of aryl halides. Trifluoroacetic anhydride (TFAA) is essential for the activation of the C–CN bond, while the exact role of KF remains unclear; however, its absence leads to a significant decrease in product yield.

**Scheme 17:**
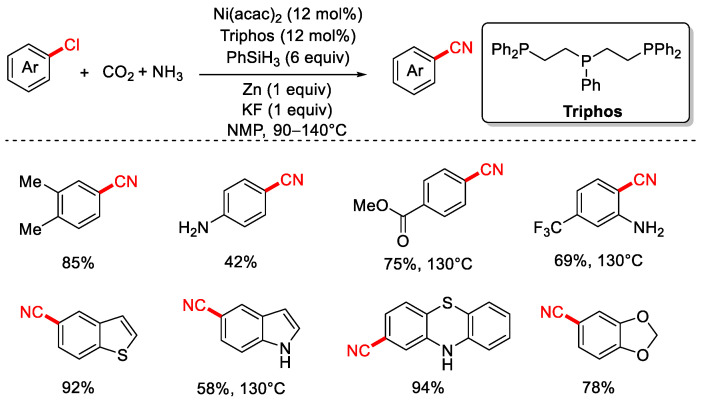
Ni-catalyzed cyanation of aryl chlorides using CO_2_/NH_3_ as a cyano source.

**Scheme 18:**
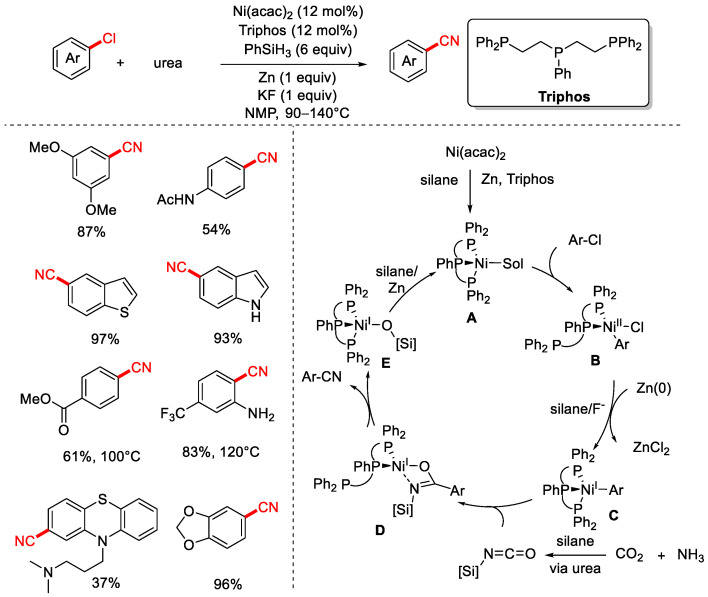
Ni-catalyzed cyanation of aryl chlorides using urea as a cyano source.

In 2020, the Li group reported a method for the cyanation of aryl chlorides using CO_2_ and NH_3_ as the cyanide source (Scheme 17) [83]. This protocol employs the tridentate phosphine ligand Triphos, PhSiH_3_ as the reductant, and Zn powder as a co-reductant, enabling nickel-catalyzed cyanation of both aryl and alkyl chlorides with high yields and good functional group tolerance. Furthermore, the group also demonstrated a series of cyanation of aryl chloride reactions using urea as the cyanide source (Scheme 18).

The proposed mechanism begins with the reduction of Ni(II) precursors to a Ni(0) species A in the presence of silanes and zinc. Next, oxidative addition of the aryl chloride to A forms a Ni(II) halide intermediate B, which is further reduced by silane and zinc to a highly nucleophilic Ni(I) intermediate C. Subsequently, silyl isocyanate formation is followed by nickel–carbon insertion, generating a presumably transient imidate species D. Further transformation proceeds via a plausible 1,3-silyl N-to-O migration to yield the cyano product, releasing a nickel siliconate intermediate E that is reduced by hydrosilane to regenerate species A (Scheme 18).

**Scheme 19:**
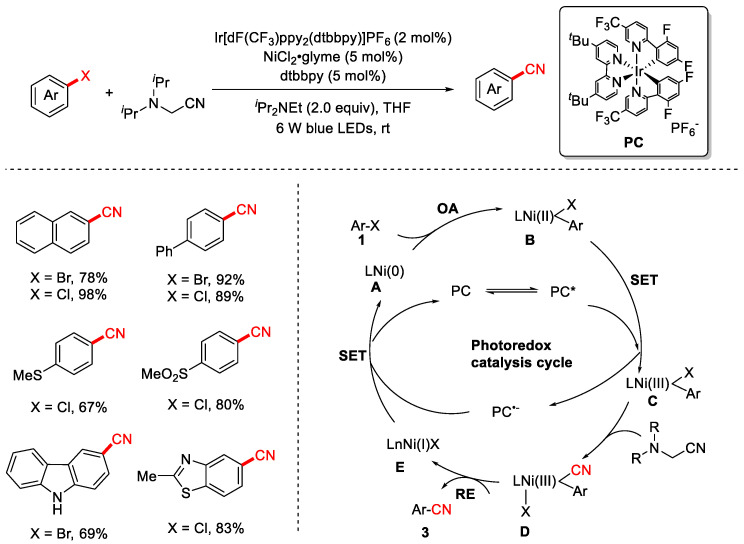
Visible-light/Ni dual-catalyzed cyanation of aryl halides.

In 2021, the Xiao group pioneered a visible-light-induced dual-catalysis strategy to achieve the cyanation of aryl halides. Under this approach, the Ni(II) species is photo-oxidized to Ni(III), thereby facilitating both cyanide transfer and catalytic turnover [84] (Scheme 19). This protocol enables the transformation to proceed under mild, room-temperature conditions using organic compounds as the cyanide source. It exhibits broad substrate scope and generally high yields. The proposed mechanism is as follows: Oxidative addition (OD) of the Ni(0) catalyst A to aryl halide 1 generates aryl–Ni(II) intermediate B. Concurrently, the ground-state photocatalyst (PC) is excited by visible light to form the excited state PC*. At this stage, a single-electron transfer (SET) from intermediate B to PC* occurs, affording the key Ni(III)–aryl species C and the reduced photocatalyst PC^−^. The Ni(III) species C subsequently accepts a cyano group from the cyanating reagent 2, generating a transient Ar–Ni(III)–CN complex D, which undergoes rapid reductive elimination (RE) to afford the desired product 3. Finally, the Ni(I) species E engages in a second SET with the reduced photocatalyst PC^−^, regenerating both the Ni(0) catalyst and the ground-state photocatalyst, thereby completing both catalytic cycles.

**Scheme 20:**
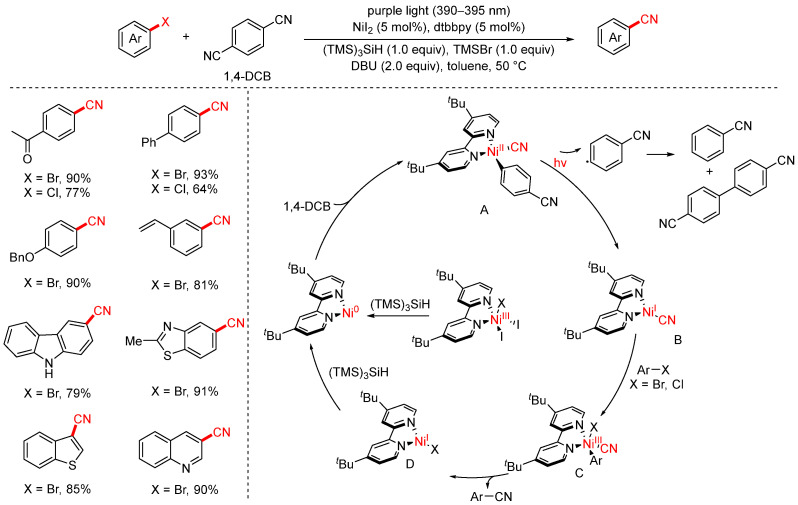
Visible-light/Ni-catalyzed cyanation of aryl halides using 1,4-dicyanobenzene.

In 2022, the Xue group developed a visible-light-promoted cyanation of aryl halides strategy using 1,4-dicyanobenzene (1,4-DCB) as the cyanation reagent (Scheme 20) [85]. This transformation proceeds under mild conditions with NiI_2_ as the catalyst, dtbbpy as the ligand, (TMS)_3_SiH as the reductant, DBU as the base, and TMSBr as an additive. Upon irradiation with purple LED light (50 °C, 16 h), a variety of aryl halides could be converted to the corresponding aryl nitriles in good to excellent yields. The addition of TMSBr was found to be crucial for suppressing the undesired hydrogenation of aryl halides. Notably, both aryl bromides and aryl chlorides are compatible, and the reaction exhibits broad substrate scope and high efficiency. The proposed mechanism is as follows: Initially, the in situ-formed Ni(II) complex is reduced by (TMS)_3_SiH to generate a Ni(0) species. This Ni(0) species undergoes oxidative addition with 1,4-DCB to form Ni(II) complex A. Upon irradiation with purple light, complex A undergoes homolytic cleavage of the Ni–C bond, generating an aryl radical and Ni(I) complex B. Subsequently, oxidative addition of the aryl halide to Ni(I) complex B gives rise to Ni(III) intermediate C, which then undergoes reductive elimination to deliver the desired aryl nitrile and regenerate Ni(I) complex D. Finally, complex D is reduced back to Ni(0), thus closing the catalytic cycle.

**Scheme 21:**
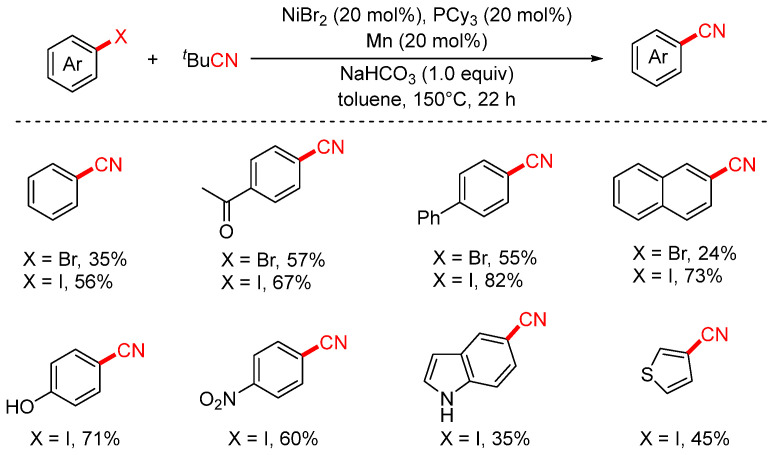
Ni-catalyzed cyanation of aryl halides using *^t^*BuCN as a cyano source.

In 2022, the Zhou group reported a nickel-catalyzed cyanation of aryl halides using tert-butyl cyanide (*^t^*BuCN) as the cyanide source (Scheme 21) [86]. The study demonstrated that both aryl iodides and aryl bromides could undergo cyanation with aliphatic isocyanides to afford aryl nitriles in moderate to good yields. This transformation features broad substrate scope and excellent functional group tolerance, accommodating a variety of substituents such as hydroxyl (–OH), nitro (–NO_2_), and aldehyde (–CHO) groups.

**Scheme 22:**
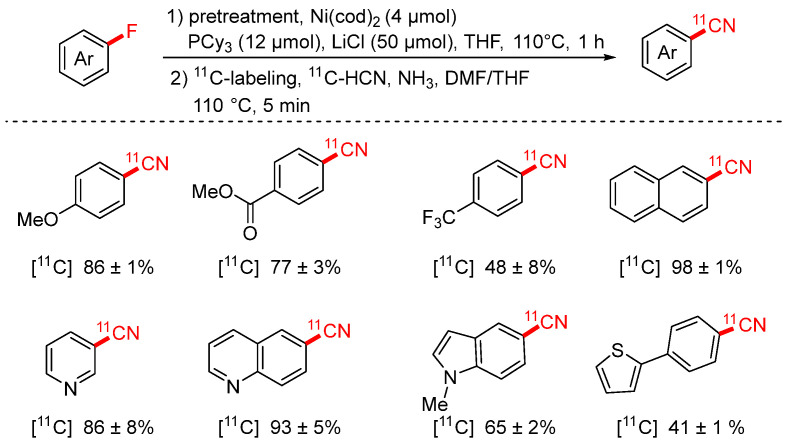
Ni-mediated [^11^C] cyanation of aryl fluorides.

In 2023, the Hosoya group reported a two-step radiosynthetic approach for the preparation of [^11^C]-labeled aryl nitriles via nickel-mediated C–F bond activation (Scheme 22) [87]. This strategy enables the in situ de-fluoro-cyanation of aryl fluorides to afford [^11^C]aryl nitriles efficiently. In the first (pretreatment) stage, the formation of the aryl–Ni(II) complex is crucial for the success of the reaction. The addition of LiCl as an additive was found to be essential in this step; replacing it with other additives led to a significant drop in product yield, highlighting its critical role in promoting Ni complex formation or stabilization.

**Scheme 23:**
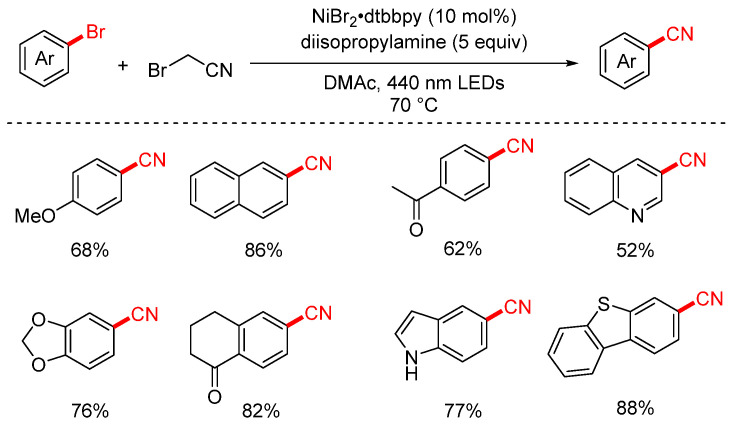
Visible-light-catalyzed cyanation of aryl halides using bromoacetonitrile.

In 2023, the Rueping group developed a visible-light-induced cyanation of aryl halides using bromoacetonitrile (BrCH_2_CN) as the cyanide source (Scheme 23) and further extended the strategy to a decarboxylative cyanomethylation reaction [88]. Remarkably, the authors discovered that the dehalogenative cyanation could proceed even in the absence of a photocatalyst, suggesting a possible self-sensitized or substrate-driven photoactivation pathway. The reaction displays broad substrate scope and delivers the desired products in generally good yields, making it a valuable and practical synthetic method.

**Scheme 24:**
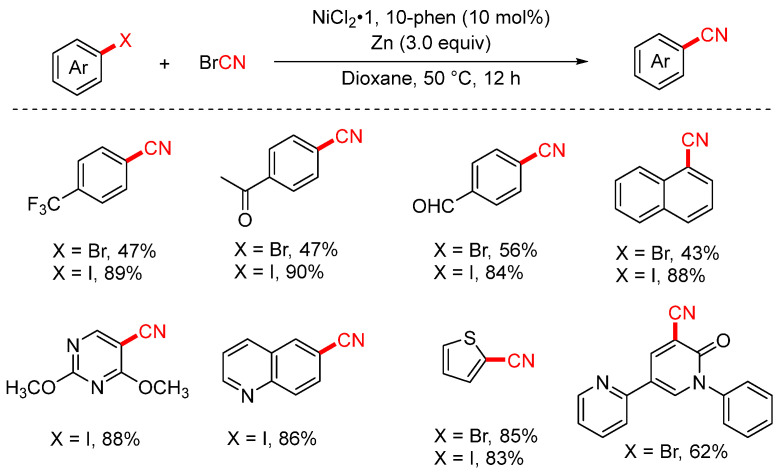
Ni-catalyzed cyanation of aryl halides using BrCN as a cyano source.

In 2024, the Liang group developed an efficient cyanation of aryl iodides and aryl bromides using commercially available cyanogen bromide (BrCN) as the cyanide source (Scheme 24) [89]. This method enables the transformation of a broad range of functionalized aryl halides into the corresponding aryl nitriles with moderate to good yields. Notably, the protocol was successfully scaled up to the gram scale, and it demonstrated excellent utility in late-stage cyanation of complex natural products and pharmaceutical molecules, underscoring its potential for practical synthetic applications.

### 3.2. Cyanating Solvents

**Scheme 25:**
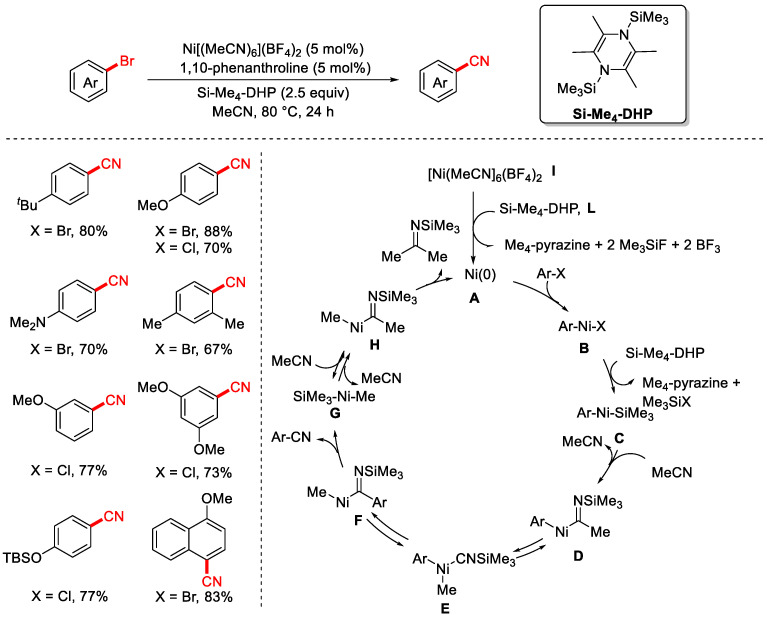
First Ni-catalyzed cyanation of aryl chlorides using acetonitrile.

In 2019, the Mashima group reported the cyanation of aryl halides using a common organic solvent, acetonitrile, as the cyanating reagent for the first time (Scheme 25) [90]. The reaction employed [Ni(MeCN)_6_](BF_4_)_2_ as the catalyst, 1,10-phenanthroline as the ligand, and Si–Me_4_-DHP as the reductant to enable efficient transformation of aryl halides into the corresponding aryl nitriles. A key highlight of this work is the novel use of MeCN itself as the cyanide source in a Ni-catalyzed cyanation reaction. In this system, Si–Me_4_-DHP functions as the reductant, reducing Ni(II) to Ni(0). The proposed reaction mechanism proceeds as follows: First, Ni(0) undergoes oxidative addition with the aryl bromide to generate a Ni(II) intermediate A. This intermediate is then reduced by zinc to form a Ni(I)–aryl species B. After undergoing a 1,2-migratory insertion with MPMN (most likely a methylene source in this context), a Ni(I)-coordinated imine intermediate C is formed. This species then undergoes β-C elimination to produce the aryl nitrile product and an α-nickelated intermediate D. Subsequent ligand exchange with ZnBr_2_ yields a Ni(I)–Br species E, which is reduced by zinc to regenerate the Ni(0) catalyst, thereby completing the catalytic cycle.

**Scheme 26:**
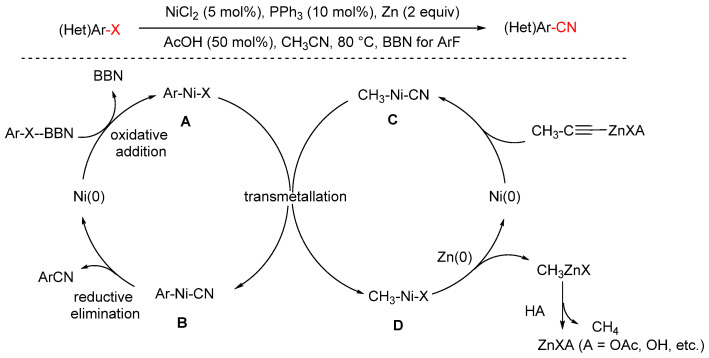
Ni-catalyzed cyanation of aryl fluorides using acetonitrile.

In 2024, the Meguellati group reported a nickel-catalyzed cyanation of aryl fluorides using low-toxicity acetonitrile (MeCN) as the cyanide source (Scheme 26) [91]. Given the high bond dissociation energy of the C–F bond, activating aryl fluorides is notoriously difficult. To address this, the authors employed OMe-BBN, a Lewis acidic borane, to facilitate C–F bond cleavage. The reaction proceeds under catalysis by NiCl_2_ with PPh_3_ as the ligand, affording the desired aryl nitrile products. Mechanistically, the authors proposed the following pathway: A Lewis acid plays a pivotal role in cleaving the C–F bond in aryl fluorides. OMe-BBN facilitates the oxidation of aryl fluorides to zerovalent nickel species, forming aryl–Ni intermediate A. Simultaneously, assisted by zinc salts, oxidative addition of acetonitrile to Ni(0) gives cyano–Ni species C. A ligand exchange between species A and C furnishes intermediate B, which undergoes reductive elimination to yield the desired aryl nitrile 2 and regenerate the Ni(0) species. Meanwhile, intermediate D, generated during transmetalation, is reduced back to Ni(0) by Zn(0), completing the catalytic cycle.

## 4. Conclusions

Nickel-catalyzed dehalogenative cyanation has become a valuable and versatile strategy for constructing aryl nitriles under mild and sustainable conditions. By employing various cyanide sources—including traditional metal cyanides and modern organic alternatives—significant progress has been made in broadening substrate scope, improving catalyst efficiency, and reducing environmental hazards. Notably, the development of air-stable catalysts, greener reductants, and photocatalytic systems has greatly enhanced the practicality and safety of these transformations. However, challenges such as limited reactivity of aryl chlorides, poor functional group compatibility in certain systems, and the need for more sustainable cyanide surrogates still remain. Continued efforts in catalyst design, mechanistic understanding, and green process development will be critical to advancing this area toward industrial applications and environmentally friendly synthesis of nitrile-containing compounds.

## Data Availability

The research data are available by contacting the corresponding author.

## Abbreviations

The following abbreviations are used in this manuscript:

Ar–X	Aryl halide
Ar–CN	Aryl nitrile
TMSCN	Trimethylsilyl cyanide
DMF	N,N-Dimethylformamide
HMPA	Hexamethylphosphoramide
PMHS	Polymethylhydrosiloxane
DABAL-Me_3_	Tris(dimethylaluminum)aluminate
TBABr	Tetrabutylammonium bromide
COD	1,5-Cyclooctadiene
DQ	1,4-Benzoquinone
MPMN	2-methyl-2-phenyl malononitrile
DMAc	N,N-Dimethylacetamide
TFAA	Trifluoroacetic anhydride
Triphos	Tris[2-(diphenylphosphino)ethyl]phosphine
PC	Photocatalyst
SET	Single-electron transfer
RE	Reductive elimination
LED	Light-emitting diode
dtbbpy	4,4′-Di-tert-butyl-2,2′-bipyridine
TMSB	Trimethylsilyl bromide
OMe-BBN	Methoxy-substituted borabicyclo[3.3.1]nonane
